# Low-Grade Inflammatory Hematological Markers in Otolaryngologic Diseases: A Preliminary Report

**DOI:** 10.3390/diseases13100328

**Published:** 2025-10-03

**Authors:** María Aurora Maravilla-Domínguez, Beatriz Teresita Martín-Márquez, Flavio Sandoval-García, Verónica Adriana Montes-Varela, Nicté Selene Fajardo-Robledo, Fernanda Isadora Corona-Meraz, Soraya Amalí Zavaleta-Muñiz

**Affiliations:** 1 Facultad de Ciencias de la Salud, Universidad Juárez del Estado de Durango, Durango 35050, Estado de Durango, Mexico; draauroramaravilla@gmail.com; 2 Cuerpo Académico UJED-CA-135, “Elementos Ambientales Nutricionales en el Proceso de Enfermedad”, Universidad Juárez del Estado de Durango, Durango 35050, Estado de Durango, Mexico; 3 Instituto de Investigación en Reumatología y del Sistema Músculo Esquelético, Departamento de Biología Molecular y Genómica, Centro Universitario de Ciencias de la Salud, Universidad de Guadalajara, Guadalajara 44340, Jalisco, Mexico; flavio.sandoval@academicos.udg.mx; 4 Cuerpo Académico UDG-CA-703, “Inmunología y Reumatología”, Centro Universitario de Ciencias de la Salud, Universidad de Guadalajara, Guadalajara 44340, Jalisco, Mexico; 5 Sanatorio San José, Gómez Palacio, Durango 35000, Estado de Durango, Mexico; 6 Laboratorio de Investigación y Desarrollo Farmacéutico, Departamento de Farmacobiología, Centro Universitario de Ciencias Exactas, Universidad de Guadalajara, Guadalajara 44430, Jalisco, Mexico; nicte.fajardo@academicos.udg.mx; 7 Centro de Investigación Multidisciplinaria en Salud, Departamento de Ciencias Biomédicas, División de Ciencias de la Salud, Centro Universitario de Tonalá, Universidad de Guadalajara, Tonalá 45425, Jalisco, Mexico; mariaf.corona@academicos.udg.mx

**Keywords:** ear, nose and throat diseases, inflammation markers, hearing loss, neutrophils, lymphocytes

## Abstract

**Background/Objectives**: Complete blood count tests are inexpensive and widely available and may help identify low-grade inflammation in otolaryngologic (Ear, Nose and Throat, ENT) diseases, such as facial paralysis and hearing loss. This study aimed to describe the neutrophil-to-lymphocyte ratio (NLR), platelet-to-lymphocyte ratio (PLR), monocyte-to-lymphocyte ratio (MLR), eosinophil-to-lymphocyte ratio (ELR), and lymphocyte-to-monocyte ratio (LMR) in ENT diseases and to provide preliminary evidence supporting further research. **Methods**: Data from 62 patients with ENT diseases were analyzed in a cross-sectional design. **Results**: The prevalence of ENT diseases was higher in women (63%) and adults (85.5%), highlighting vertigo, hearing loss, and septal deviation. Most marker values were within normal ranges; however, NLR values were elevated in patients with either septal deviation or vertigo, and ELR values were increased in cases of allergic or infectious rhinitis and sinusitis. In contrast, LMR values were at the lower normal limits in patients with septal deviation. **Conclusions:** These findings highlight the need for further studies to clarify the role of these biomarkers in chronic conditions and morphological alterations associated with ENT diseases, using more complex study designs.

## 1. Introduction

Ear, nose, and throat (ENT) disorders, addressed by the medical specialty of otolaryngology, represent a common and significant health concern worldwide. ENT-related complaints represent a substantial proportion of medical consultations, reaching 49% in some reports. They affect 52.2% of pediatric patients who visit emergency departments with ENT specialist-related diagnoses, highlighting the relevance of research in this field [1,2]. The high prevalence of rhinitis, vertiginous disorders, and infectious diseases as the most common reasons for consultation of ENT pathologies in Colombia between 2015 and 2019 underscores the urgency for ongoing research in this area. The highest prevalence age groups are children aged 1 to 5 years, with a second peak occurring between 27 and 44 years [3]. In a specialized hospital in Spain, Tenor-Serrano et al. reported that 18.3% of patients had hearing loss, 9.2% experienced nasal breathing difficulties, and 7.6% suffered from dizziness [4].

In Mexico, the lack of comprehensive epidemiological studies on ENT diseases highlights the pressing need for further research. In 2009, studies conducted by Chávez et al. reported that, by anatomical region, 43% of patients were referred for issues related to the nose and paranasal sinuses, 38% were referred for the pharynx, 17% were referred for the ear, and 2% were referred for the larynx. By pathology, the most common conditions were chronic tonsillitis, septal deviation, and chronic pharyngitis (26%, 23%, and 13%, respectively) [5]. Xacur-Hernández et al. reported that the three leading causes of ENT diseases requiring hospital admission in 2015 were chronic tonsillitis, nasal septum deviation, and unspecified acute pharyngitis [6].

All ENT diseases share a common inflammatory process that can alter hematological inflammatory markers. Recently, elevated values of ratios such as the neutrophil-to-lymphocyte ratio (NLR), monocyte-to-lymphocyte ratio (MLR), and platelet-to-lymphocyte ratio (PLR) have been reported [1]. Elevated NLR values have been reported in patients with sudden sensorineural hearing loss [7,8], particularly in bilateral cases [9], as well as in Bell’s palsy [10], tinnitus [11], and severe idiopathic peripheral facial paralysis [12].

This study’s findings on the potential of NLR and PLR as prognostic markers for sudden sensorineural hearing loss could encourage new research and clinical approaches, potentially improving patient outcomes and quality of life, offering hope for better management of this condition [2]. Given this background, our study is the first to investigate the status of inflammatory markers in patients with otolaryngological conditions in Mexico. This unique aim sets our research apart and significantly contributes to understanding these conditions, potentially paving the way for more targeted and effective treatments.

## 2. Materials and Methods

### 2.1. Patients

We conducted a cross-sectional study including all patients from the outpatient Otolaryngology department of a secondary-care hospital during the first six months of the year, provided they had a complete clinical record and complete blood count results. We rigorously excluded subsequent patients and those lacking complete results. Collected variables included sociodemographic data, consultation reasons, allergy and chronic disease history, signs and symptoms, laboratory tests, and final specialist diagnosis.

This study was conducted in accordance with the principles of the Declaration of Helsinki. Under the General Health Law in Health Research, the research was classified as Category I, a designation indicating that it posed no risk to participants. The protocol underwent a rigorous approval process by the Ethics and Research Committee of the Faculty of Health Sciences of the Juarez University of the State of Durango, with approval folio 2-24 and registration number PI2024-05-01, respectively. It was approved on 2 October 2024.

### 2.2. Hematological Parameters

Red blood cells, white blood cells, neutrophils, monocytes, lymphocytes, and platelet counts were performed using standard routine laboratory methods. To evaluate inflammatory hematological ratios from the complete blood count, the following calculations were performed: NLR was obtained by dividing the neutrophil count by the lymphocyte count, providing a measure of the body’s immune response. PLR was obtained by dividing the platelet count by the lymphocyte count, providing insights into the body’s inflammatory state. MLR was obtained by dividing the monocyte count by the lymphocyte count, indicating the body’s immune regulation. ELR was obtained by dividing the eosinophil count by the number of lymphocytes, and LMR was obtained by dividing the lymphocyte count by the number of monocytes. Cut-off values were defined based on previously published studies [13,14] or statistical calculations from our patient population.

### 2.3. Statistical Analysis

For statistical analysis, frequencies and percentages were used for categorical variables, and measures of central tendency, such as means and standard deviations (SD) or medians and quartiles 1 and 3, were employed for quantitative variables, depending on the normality of the data. The analyses were performed in SPSS, version 25 (IBM SPSS Statistics, Chicago, IL, USA).

## 3. Results

### 3.1. Patients’ General Demographic and Clinical Characteristics

Our comprehensive study, which involved 62 patients attending the ENT outpatient department for the first time, presents specific characteristics. For instance, we found that adult patients (aged 18 years and above) were the most common attendees, with females being the predominant group. Table 1 presents the thorough and detailed characteristics of the patients in our study.

### 3.2. Main Reasons for Referral and Diagnoses of the ENT Specialist

The primary referral reasons in the pediatric population (age < 18 years) were hearing loss, epistaxis, and tonsillar hypertrophy. Among the primary diagnoses made by the specialist, notable findings included hearing loss (24.2%), septal deviation (19.4%), vertigo (14.5%), allergic rhinitis (9.7%), and infectious rhinitis or sinusitis (8.1%). Other diagnoses included tonsillar or turbinate hypertrophy and facial paralysis.

The five most prevalent symptoms were hearing loss, nasal obstruction, tinnitus, dizziness, posterior discharge, and rhinorrhea. It is important to highlight a significant delay of at least one month between the consultation with the family doctor and the appointment with the otolaryngology specialist. This highlights the pressing need for more efficient referral and scheduling processes, which can substantially enhance patient outcomes.

In children, turbinate/tonsillar hypertrophy and hearing loss remain the most prevalent specialist diagnoses, with symptoms including hearing loss (55.5%), epistaxis (33.3%), nasal crusts (22.2%), and otalgia (22.2%). For adults, the most frequent diagnoses are hearing loss, septal deviation, and vertigo, with a diverse range of symptoms including hearing loss (52.8%), nasal obstruction (41.5%), tinnitus (41.5%), dizziness (41.5%), posterior discharge (41.5%), rhinorrhea (32.1%) and sensation of blocked ears (32.1%).

Table 2 and Table 3 present the primary reasons for referral and the primary diagnoses of an ENT specialist, highlighting the complexity and variety of these conditions.

### 3.3. Inflammatory Hematological Markers

The inflammatory hematological ratios were calculated as described in Section 2. Threshold values were derived either from previously published studies or from our patient population, as no reference values are available for healthy Mexican individuals. Overall, the mean values for NLR, MLR, LMR, and PLR were within the calculated reference ranges derived from our study population and previously published literature for MLR and ELR.

Cut-off values for NLR, PLR, and LMR were determined based on the study sample. Specifically, for NLR and PLR, the arithmetic mean plus two standard deviations (1.65 and 132.4, respectively), yielding cut-off values of 3.23 for NLR and 219.76 for PLR. For LMR, the arithmetic mean (3.63) minus two standard deviations (1.95) resulted in a cut-off value of 1.68. For MLR (≥0.38) and ELR (≥0.07), previously published studies by Liao et al. and Bedolla et al. were used [13,14].

All complete blood count parameters were within the age-specific reference ranges established for different age groups. The calculated inflammatory ratios (NLR, MLR, ELR, and PLR) are summarized in Table 4.

While mean values remained within calculated reference ranges, a subset of patients exhibited elevated markers: 11.3% had NLR > 3.23, 6.6% had PLR > 219, 6.6% had MLR > 0.038, 50.8% had ELR > 0.07, and 4.9% had low LMR (<3).

These findings, despite the average values being normal, highlight a significant subset of patients with abnormal marker levels.

The findings of this research have the potential to impact clinical practice significantly. ELR was found to be elevated in 72% of patients with allergic rhinitis, infectious rhinitis, or sinusitis, while an elevated NLR was observed in 42% of patients with tonsillar or turbinate hypertrophy. These discoveries could lead to more accurate and timely diagnosis and treatment strategies, underscoring the urgency and importance of these findings for healthcare professionals.

Our research’s comprehensive categorization of conditions—into ear (hearing loss/vertigo), nose and paranasal sinuses (rhinitis/sinusitis), tonsillar or turbinate hypertrophy, and anatomical defects of the nose (septal deviation)—demonstrates the thoroughness of our approach. While the average values of all markers remained within reference ranges, we found specific elevations in certain groups. For instance, an elevated NLR and ELR were found in 42.8% of patients with tonsillar or turbinate hypertrophy. Additionally, an elevated ELR was observed in the hearing loss/vertigo, septal deviation, and rhinitis/sinusitis groups (45.8%, 50%, and 72.7%, respectively). These findings, summarized in Table 5, provide a robust basis for further research and clinical applications, instilling confidence in their validity and reliability.

## 4. Discussion

ENT pathologies may account for up to 49% of general consultations, as noted by a previous study [1]. This current study, conducted at a secondary care center, highlights the prevalence of common pathologies, including hearing loss, septal deviation, and vertigo. Stratification by age group revealed that in patients under 18 years old, the most frequent pathologies were tonsillar hypertrophy (40%) and hearing loss (30%). In patients over 18, the most common pathologies were hearing loss (23.1%), septal deviation (21.2%), and vertigo (17.3%). Notably, although there are no records of the frequency of pediatric Otolaryngology consultations in Family Medicine, pediatric patients (<18 years old) represented less than 15% of our study population. This suggests that more pediatric cases may require specialist evaluation after resolution of acute episodes.

Hearing loss is not just a local concern; it affects people worldwide. However, a significant global health issue, affecting around 500 million people worldwide (5.8% of the global population), is one of the most common sensory disorders [15]. In the United States, for instance, over 10% of individuals experience some degree of hearing loss that affects daily communication [15]. According to the World Health Organization, the global prevalence of hearing loss is estimated at over 5% of the population (approximately 430 million people). In contrast, the prevalence observed in our study population was 24%, markedly higher than the global estimate [4]. The most common symptoms among these patients were tinnitus, a sensation of blocked ears, and imbalance. This symptomology suggests that some patients may have been experiencing this condition for a prolonged period.

Septum deviation, an anatomical defect characterized by misalignment with other structures, is a highly prevalent condition [16]. While it is often asymptomatic and not considered a pathological condition, it is the leading cause of nasal obstruction [17]. Our study’s findings underscore the importance of this condition, showing that a significant 83% of patients with septal deviation experienced nasal obstruction.

Vertigo, a condition affecting approximately 20–30% of individuals at some point in their lives [18], had a prevalence of 14% in our study. In 88.9% of these cases, patients reported a sensation of movement or spinning. These findings offer valuable insights for medical professionals, particularly those treating allergic rhinitis, where the most common symptoms are rhinorrhea and nasal obstruction, as reported by Bousquet and colleagues [19]. Our data align closely with these reports, showing that the most frequent symptoms included nasal obstruction, posterior nasal discharge, headache, and dysgeusia.

In Mexico, the role of the treating physician in the diagnostic process is of utmost importance. When a patient is referred to a specialist in secondary care, general laboratory tests, such as a blood count, are frequently requested for the initial evaluation. The treating physician’s reliance on these accessible and affordable tests to predict complications and establish a prognosis underscores their significant responsibility in patient care.

There is a growing interest in the use of inflammatory hematological indices such as NLR, PLR, and MLR. Among these, NLR has been extensively studied and is considered a marker of subclinical inflammation. It has been identified as a novel parameter for various conditions, including facial paralysis, heart disease, and several types of malignancy. Elevated NLR, MLR, and PLR biomarkers have been associated with ENT conditions, including idiopathic sensorineural hearing loss and allergic rhinitis in pediatric patients [20,21].

The potential of NLR, MLR, and PLR in managing ENT conditions is significant, highlighting their practical value in patient care.

The NLR, a key inflammatory marker, is known to be elevated in patients with idiopathic peripheral facial paralysis. This finding underscores the importance of NLR in diagnosing and managing this condition. Additionally, there is evidence of increased NLR in patients with Bell’s palsy [10] and tinnitus [11]. Moreover, NLR, MLR, and PLR are elevated in patients with idiopathic sudden sensorineural hearing loss, suggesting their potential role in identifying and managing this condition, especially in those experiencing bilateral issues [9].

Patients with allergic rhinitis display increased eosinophil counts, a higher leukocyte-to-lymphocyte ratio (L/LR), and a higher NLR. Notably, NLR and PLR have been identified as potent diagnostic markers of severity in pediatric patients, highlighting their potential to forecast disease progression [20,21]. These inflammation markers are gaining importance in evaluating inflammation, categorizing severity, and predicting the onset of complications in ENT diseases [22]. Nevertheless, the hierarchical structure of the Mexican health system may pose a risk factor for the advancement of complications, including hearing loss [23], which is often linked with adenoid hypertrophy in children and was found to have a high prevalence in the adult population in this study.

Consequently, these markers could serve as prognostic indicators of hearing loss in patients with adenoid hypertrophy and chronic otitis. They could also be beneficial for the classification and staging of rhinitis. In studies focused on other diseases (renal cancer, patients with chronic kidney disease on hemodialysis, and other types of cancer), elevated values have been established for NLR > 3, PLR > 200 [24,25], and MLR > 0.38 [13], similar to those found in this study (>3.23 for NLR and >219.76 for PLR).

Although numerous studies have examined these markers, few have addressed their importance in the pathophysiology of non-communicable diseases [26,27]. To date, there are no established reference values for healthy controls in Mexico with which to compare the results obtained in this study. This absence represents a significant gap in our knowledge and a potential barrier to adequate healthcare. However, the prospect of establishing reference values in the healthy Mexican population, as well as their role as risk and prognostic factors, could mark the beginning of a new era of precision medicine, offering a promising future with improved healthcare outcomes. This could help identify individuals whose symptoms progress to a severe level or lead to significant complications, such as sensory impairment (hearing loss), which significantly diminishes the quality of life and social adaptation. Therefore, it is crucial to establish these reference values, compare them with the levels observed in the different pathologies addressed in this study, and conduct further research with more complex designs that will enhance our understanding of these markers in these patients.

Despite its limitations, this study presents findings that are significant. The small number of participants, limited to first-time patients in the Otolaryngology department, and the delay between referral and consultation are notable. However, the potential benefits of using inflammatory markers are substantial and offer hope for the future of healthcare. These markers, easily accessible and inexpensive, can be a valuable tool that can be utilized at all levels of healthcare. They are not only readily available but also offer a promising avenue for further research and application in our own work.

In Mexico, where access to care is limited and poverty is a growing issue, the use of these markers can empower physicians to diagnose and establish prognoses for various diseases, providing reassurance in resource-limited settings. On the other hand, several methodological and contextual factors warrant careful consideration: selection bias arising from the non-random recruitment of participants, the absence of contemporaneous healthy controls, potential confounding by comorbidities that may alter marker levels, and questions about the generalizability of the results beyond the study cohort to other populations and settings. These limitations, while significant, do not diminish the potential of inflammatory markers in healthcare.

Future research must urgently address the limitations of this study to establish the utility of the markers across diverse Mexican populations and, more broadly, in similar resource-limited settings. While this study lays the groundwork, careful interpretation and continued investigation are essential to translate these promising insights into reliable, widely applicable clinical tools.

## 5. Conclusions

ELR was the most significantly altered biomarker in ENT diseases, while NLR was particularly elevated in patients with tonsillar or turbinate hypertrophy. Further validation studies are essential to confirm these findings. Investigating the behavior of these biomarkers in chronic ENT diseases and in relation to morphological changes using more complex designs is not only relevant but essential to pave the way for future research that can build on these findings.

## Figures and Tables

**Table 1:** Demographic and clinical characteristics of the patients.

Characteristic	Total (*n* = 62) *n* (%)	<18 Years (*n* = 9) *n* (%)	≥18 Years (*n* = 53) * n (%)
Age (years), mean ± SD	47.2 ± 21.5	9.1 ± 3.5	53.6 ± 1.6
Female	39 (62.9)	4 (44.4)	35 (66.0)
Family history of allergies	9 (14.5)	3 (33.3)	6 (11.3)
Personal history of allergies	22 (35.5)	3 (33.3)	19 (35.8)
Drug allergy	13 (21.0)	2 (22.2)	11 (20.8)
Food allergy	2 (3.2)	1 (11.1)	1 (1.9)
Animal allergy	6 (9.7)	1 (11.1)	5 (9.4)
Personal history of diabetes	18 (29.0)	0	18 (34.0)
Personal history of hypertension	18 (29.0)	0	18 (34.0)
Personal history of dyslipidemia	16 (25.8)	0	16 (30.2)

* Note: Only 5% of patients ≥ 18 years underwent immunotherapy treatment. SD: standard deviation.

**Table 2:** Main reasons for referral to ENT specialist.

Reason for Referral	Total (*n* = 62) *n* (%)	<18 Years (*n* = 9) *n* (%)	≥18 Years (*n* = 53) *n* (%)
Septal deviation	4 (6.4)	0	4 (7.6)
Hearing loss	13 (20.9)	3 (33.3)	10 (19.0)
Epistaxis	2 (3.2)	2 (22.2)	0
Rhinitis/sinusitis/otitis	17 (27.4)	0	17 (32.1)
Vertigo	14 (22.5)	0	14 (26.6)
Turbinate/tonsillar hypertrophy	4 (6.4)	2 (22.2)	2 (3.8)
Facial paralysis	1 (1.6)	1 (11.1)	0
Dysphonia/cordal paralysis	2 (3.2)	0	2 (3.8)
Other (tumor, joint pain, tinnitus)	3 (4.8)	0	0

Note: One of the total subjects (1.6%) was referred for combined pathology (hearing loss/allergic rhinitis), 1 (1.6%) was referred for tinnitus, and 1 (1.6%) was referred for dysphonia; others were referred for a tumor or joint pain.

**Table 3:** Main diagnoses made by ENT specialist.

Diagnosis	Total (*n* = 62) *n* (%)	<18 Years (*n* = 9) *n* (%)	≥18 Years (*n* = 53) *n* (%)
Septal deviation	12 (19.4)	0	12 (22.6)
Hearing loss	15 (24.2)	3 (33.3)	12 (22.6)
Allergic rhinitis	6 (9.7)	0	6 (11.3)
Infectious rhinitis/sinusitis	5 (8.1)	1 (11.1)	4 (7.5)
Vertigo	9 (14.5)	0	9 (17.0)
Turbinate/tonsillar hypertrophy	7 (11.3)	4 (44.4)	3 (5.7)
Facial paralysis	2 (3.2)	1 (11.1)	1 (1.9)

All data are presented as frequencies and percentages.

**Table 4:** Inflammatory markers in ENT patients.

Inflammatory Markers	<18 Years (*n* = 9) Median (Q1–Q3)	≥18 Years (*n* = 53) Median (Q1–Q3)
NLR	1.19 (0.67–1.85)	1.53 (1.15–2.0)
MLR	0.26 (0.14–0.34)	0.22 (0.17–0.29)
ELR	0.08 (0.03–0.12)	0.07 (0.04–0.11)
PLR	115.8 (67.5–141.7)	104.3 (84.8–125.3)
LMR	3.81 (2.9–7.3)	4.6 (3.5–5.7)

NLR: neutrophil/lymphocyte ratio; MLR: monocyte/lymphocyte ratio; ELR: eosinophil/lymphocyte ratio; PLR: platelet/lymphocyte ratio; LMR: lymphocyte/monocyte ratio.

**Table 5:** Inflammatory hematological ratios by diagnosis group.

Inflammatory Markers	Total *n* = 62	Hearing Loss/Vertigo *n* = 32	Septal Deviation *n* = 12	Allergic/Infectious Rhinitis or Sinusitis *n* = 11	Tonsillar/Turbinate Hypertrophy *n* = 7
Elevated NLR	7 (11.3)	1 (4.1)	2 (16.6)	0	3 (42.8)
Elevated MLR	4 (6.6)	2 (8.3)	1 (8.3)	0	2 (28.6)
Elevated ELR	31 (50.8)	11 (45.8)	6 (50.0)	8 (72.7)	3 (42.8)
Elevated PLR	4 (6.6)	3 (12.5)	1 (8.3)	0	1 (14.3)
Low LMR	3 (4.9)	2 (8.3)	0	0	2 (28.6)

NLR: neutrophil/lymphocyte ratio; MLR: monocyte/lymphocyte ratio; ELR: eosinophil/lymphocyte ratio; PLR: platelet/lymphocyte ratio; LMR: lymphocyte/monocyte ratio. Some of the patients had two or more diagnoses made by the specialist, so altered markers for both pathologies are presented.

## Data Availability

The original contributions presented in this study are included in the article. Further inquiries can be directed to the corresponding authors.

## Abbreviations

The following abbreviations are used in this manuscript:

ENT	Ear, nose, and throat diseases (otolaryngological diseases)
NLR	Neutrophil/lymphocyte ratio
PRL	Platelet/lymphocyte ratio
MLR	Monocyte/lymphocyte ratio
LMR	Lymphocytes/monocyte ratio
ERL	Eosinophil/lymphocyte ratio

## References

[1] Diaz Ledesma L., Davila Acosta J.H., Rodriguez Salazar V.M., Añaños Flores D.M. (2003). Frecuencia de diagnósticos de la especialidad de otorrinolaringología en el consultorio de medicina general en un centro de salud. Rev. Medica Hered..

[2] Signorelli L.G., Mendes Ede A. (2013). Prevalence of otorhinolaryngologic diagnoses in the pediatric emergency room. Int. Arch. Otorhinolaryngol..

[3] Ruz G.S., Breinbauer K.H., Arancibia S.M. (2009). Análisis epidemiológico de la patología otorrinolaringológica ambulatoria en el Hospital San Juan de Dios. Rev. Otorrinolaringol. Y Cirugía Cabeza Y Cuello.

[4] Tenor-Serrano R., De La Plata-Sánchez C., Colomo-Rodríguez N., Conde-Jiménez M., Oliva-Domínguez M. (2016). Motivos de consulta de pacientes atendidos en un servicio de ORL en un hospital de segundo nivel. Rev. ORL.

[5] Chávez-Ramirez G. (2009). Causas de referencia a la consulta de Otorrinolaringología, en un hospital de segundo nivel. Rev. Hosp. Juárez México.

[6] Xacur-Hernández M.I., Santos-Zaldívar K.P., Quintana-Gamboa A.A., Méndez-Domínguez N. (2020). Descripción y análisis clínico-epidemiológico de los motivos otorrinolaringológicos de ingreso en 2015 en México. An. Otorrinolaringol. Mex..

[7] Ni W., Song S.P., Jiang Y.D. (2021). Association between routine hematological parameters and sudden sensorineural hearing loss: A meta-analysis. J. Otol..

[8] Ha R., Lim B.W., Kim D.H., Park J.W., Cho C.H., Lee J.H. (2019). Predictive values of neutrophil to lymphocyte ratio (NLR), platelet to lymphocyte ratio (PLR), and other prognostic factors in pediatric idiopathic sudden sensorineural hearing loss. Int. J. Pediatr. Otorhinolaryngol..

[9] Zhang X., Weng Y., Xu Y., Xiong H., Liang M., Zheng Y., Ou Y. (2019). Selected Blood Inflammatory and Metabolic Parameters Predicted Successive Bilateral Sudden Sensorineural Hearing Loss. Dis. Markers.

[10] Kum R.O., Yurtsever Kum N., Ozcan M., Yilmaz Y.F., Gungor V., Unal A., Ciliz D.S. (2015). Elevated neutrophil-to-lymphocyte ratio in Bell’s palsy and its correlation with facial nerve enhancement on MRI. Otolaryngol. Head Neck Surg..

[11] Ozbay I., Kahraman C., Balikci H.H., Kucur C., Kahraman N.K., Ozkaya D.P., Oghan F. (2015). Neutrophil-to-lymphocyte ratio in patients with severe tinnitus: Prospective, controlled clinical study. J. Laryngol. Otol..

[12] Kilickaya M.M., Tuz M., Yariktas M., Yasan H., Aynali G., Bagci O. (2015). The Importance of the Neutrophil-Lymphocyte Ratio in Patients with Idiopathic Peripheral Facial Palsy. Int. J. Otolaryngol..

[13] Liao J., Wei D., Sun C., Yang Y., Wei Y., Liu X. (2022). Prognostic value of the combination of neutrophil-to-lymphocyte ratio, monocyte-to-lymphocyte ratio and platelet-to-lymphocyte ratio on mortality in patients on maintenance hemodialysis. BMC Nephrol..

[14] Bedolla-Barajas M., Morales-Romero J., Hernandez-Colin D.D., Larenas-Linnemann D., Mariscal-Castro J., Flores-Razo M.M., Bedolla-Pulido A. (2022). Beyond eosinophilia: Inflammatory patterns in patients with asthma. J. Asthma.

[15] Wilson B.S., Tucci D.L., Merson M.H., O’Donoghue G.M. (2017). Global hearing health care: New findings and perspectives. Lancet.

[16] Ramírez-Oropeza F., Mondragón-Angeles M., Galarza-Lozano D., Heras-Gómez D. (2009). Sutura en polea para el tratamiento de las desviaciones caudales septales. An. Otorrinolaringol. Mex..

[17] Fettman N., Sanford T., Sindwani R. (2009). Surgical management of the deviated septum: Techniques in septoplasty. Otolaryngol. Clin. N. Am..

[18] Petri M., Chirila M., Bolboaca S., Cosgarea M. (2015). Unilateral peripheral vestibular disorders in the emergency room of the ENT Department of Cluj-Napoca, Romania. Clujul Med..

[19] Bousquet J., Khaltaev N., Cruz A.A., Denburg J., Fokkens W.J., Togias A., Zuberbier T., Baena-Cagnani C.E., Canonica G.W., van Weel C. (2008). Allergic Rhinitis and its Impact on Asthma (ARIA) 2008 update (in collaboration with the World Health Organization, GA(2)LEN and AllerGen). Allergy.

[20] Cansever M., Sari N. (2022). The association of allergic rhinitis severity with neutrophil-lymphocyte and platelet-lymphocyte ratio in children. North Clin. Istanb..

[21] Kant A., Terzioglu K. (2021). Association of severity of allergic rhinitis with neutrophil-to-lymphocyte, eosinophil-to-neutrophil, and eosinophil-to-lymphocyte ratios in adults. Allergol. Immunopathol..

[22] Wu Z., Cao Y., Liu Z., Geng N., Pan W., Zhu Y., Shi H., Song Q., Liu B., Ma Y. (2024). Study on the predictive value of laboratory inflammatory markers and blood count-derived inflammatory markers for disease severity and prognosis in COVID-19 patients: A study conducted at a university-affiliated infectious disease hospital. Ann. Med..

[23] Rodriguez-Valero M., Pastolero A., Redfield S., Medrano A., Abreu-Gonzalez M., Gallardo-Ollervides J.F., Cisneros Lesser J.C., Hinojosa Valencia M.F., Poe D., Shearer E. (2024). High prevalence of syndromic hearing loss in Mexican children undergoing cochlear implantation. Laryngoscope Investig. Otolaryngol..

[24] Chrom P., Stec R., Semeniuk-Wojtas A., Bodnar L., Spencer N.J., Szczylik C. (2016). Fuhrman Grade and Neutrophil-To-Lymphocyte Ratio Influence on Survival in Patients with Metastatic Renal Cell Carcinoma Treated with First-Line Tyrosine Kinase Inhibitors. Clin. Genitourin. Cancer.

[25] Catabay C., Obi Y., Streja E., Soohoo M., Park C., Rhee C.M., Kovesdy C.P., Hamano T., Kalantar-Zadeh K. (2017). Lymphocyte Cell Ratios and Mortality among Incident Hemodialysis Patients. Am. J. Nephrol..

[26] Madjid M., Fatemi O. (2013). Components of the complete blood count as risk predictors for coronary heart disease: In-depth review and update. Tex. Heart Inst. J..

[27] Al-Hadlaq S.M., Balto H.A., Hassan W.M., Marraiki N.A., El-Ansary A.K. (2022). Biomarkers of non-communicable chronic disease: An update on contemporary methods. PeerJ.

